# Rapid biotic homogenization of marine fish assemblages

**DOI:** 10.1038/ncomms9405

**Published:** 2015-09-24

**Authors:** Anne E. Magurran, Maria Dornelas, Faye Moyes, Nicholas J. Gotelli, Brian McGill

**Affiliations:** 1 Centre for Biological Diversity and Scottish Oceans Institute, School of Biology, University of St Andrews, St Andrews, Fife KY16 9TH, Scotland, UK; 2 Department of Biology, University of Vermont, Burlington, Vermont 05405, USA; 3 School of Biology and Ecology, Sustainability Solutions Initiative, University of Maine, Orono, Maine 04469, USA

## Abstract

The role human activities play in reshaping biodiversity is increasingly apparent in terrestrial ecosystems. However, the responses of entire marine assemblages are not well-understood, in part, because few monitoring programs incorporate both spatial and temporal replication. Here, we analyse an exceptionally comprehensive 29-year time series of North Atlantic groundfish assemblages monitored over 5° latitude to the west of Scotland. These fish assemblages show no systematic change in species richness through time, but steady change in species composition, leading to an increase in spatial homogenization: the species identity of colder northern localities increasingly resembles that of warmer southern localities. This biotic homogenization mirrors the spatial pattern of unevenly rising ocean temperatures over the same time period suggesting that climate change is primarily responsible for the spatial homogenization we observe. In this and other ecosystems, apparent constancy in species richness may mask major changes in species composition driven by anthropogenic change.

## Abstract (web summary)

The response of marine fish assemblages to global change is not fully understood. Analysing a 29-year time-series, Magurran *et al.* show that despite little change in species richness, high species turnover is leading to North Atlantic groundfish assemblages becoming spatially homogenized, likely as a result of climatic change.

## Graphical abstract

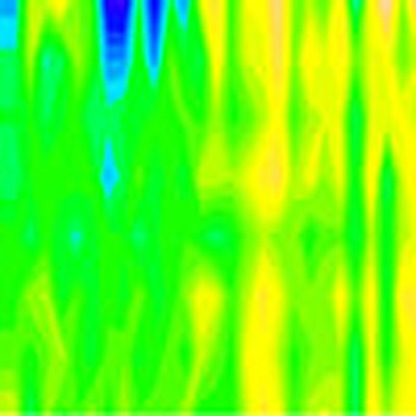

The Anthropocene^1^,^2^ is characterized by marked changes in the diversity of natural assemblages across the Earth^3^. There is, however, increasing evidence that community responses to anthropogenic impacts are complex and scale dependent^4^. Although heavily transformed terrestrial habitats exhibit species loss^3^, recent meta-analyses have detected no systematic change in local α diversity^5^,^6^. In contrast, temporal turnover (temporal β diversity)^7^ in local assemblages is occurring at rates in excess of background levels predicted by null and neutral models^6^. β diversity measures change in community composition over time (or space), and will be influenced by a range of processes including species invasion and shifts in species ranges. Dornelas *et al.*^6^ hypothesized that elevated rates of turnover could be a result of increasing biotic homogenization, a process whereby previously differentiated assemblages increasingly resemble one another in species composition. This hypothesis of a linkage between untrending local species richness, high turnover in local species composition and declining spatial β diversity (increasing homogenization) has not yet been tested in any ecosystem.

To date, investigations of biotic homogenization have focused on direct human influences on freshwater and terrestrial assemblages^8^. For example, deliberate introductions of gamefish are largely responsible for the greater homogeneity of contemporary freshwater fish assemblages in the United States compared with the time of the European settlement^9^. However, open water marine communities–especially those in offshore localities–are arguably less vulnerable to biotic homogenization than terrestrial ones because they are initially less spatially differentiated^10^,^11^,^12^ and less subject to some of the direct human interventions driving terrestrial homogenization such as species introductions and habitat modification. Nonetheless, for many marine species^13^, recent shifts in geographic ranges and phenology have been linked to anthropogenic climate change^14^,^15^,^16^,^17^ and temporal turnover in marine assemblages has also increased relative to null expectations^6^. This makes offshore marine ecosystems a good candidate for testing the role of elevated community turnover in homogenization.

Using data collected during a systematic Scottish groundfish survey, we probe marine community shifts by quantifying temporal trends in α and β diversity over almost three decades. We show that biotic homogenization is driven by elevated species turnover, transforming marine assemblages in ways that resemble terrestrial assemblages.

## Results

### α and β diversity

**Figure 1:**
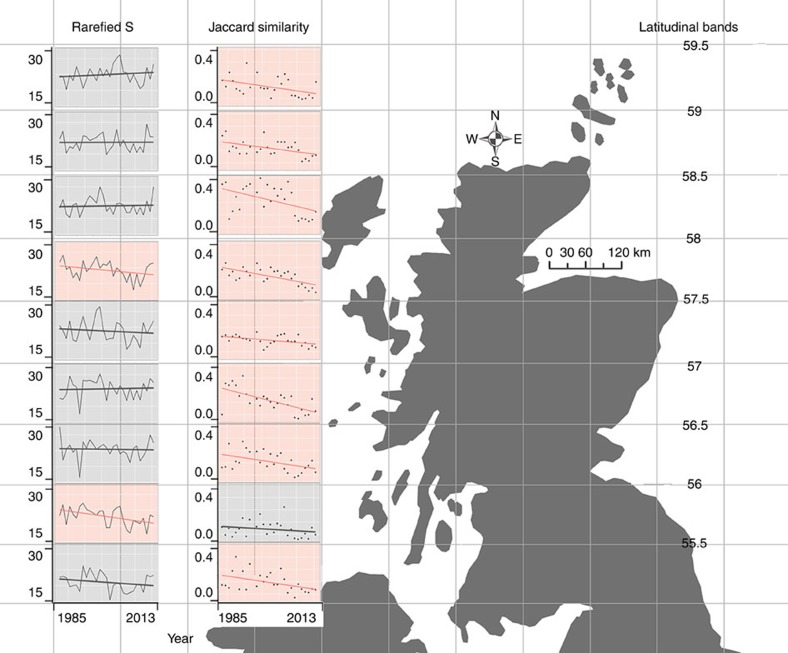
Temporal trends in α diversity (rarefied species richness) and β diversity (Jaccard similarity, relative to the initial year), in each latitudinal band, over the duration of study. Trend lines (OLS regression) are colour coded red if significantly negative (*P*<0.05, *n*=28) and grey if not significant. (There were no significant positive slopes). See Supplementary Figure 2 for overall trend.

With a total regional count of 131 species recorded, and an average local species richness of 21.5 (per 30 latitudinal band, per year), there was no systematic change in rarefied species richness across the 29 years of sampling (ordinary least squares (OLS) regression, *r*^2^=0.005, *P*=0.15, *n*=252, see Supplementary Figure 2), although the nine 30′ latitudinal bands exhibit heterogeneous temporal trends in species richness (α diversity; left-column panels, Fig. 1). In contrast to species richness, community composition shows increasing change relative to the baseline level within each latitudinal band, reflecting the temporal turnover that is typical for other marine, terrestrial and freshwater assemblages^6^ (Jaccard similarity; right-column panels, Fig. 1).

### Biotic homogenization

**Figure 2:**
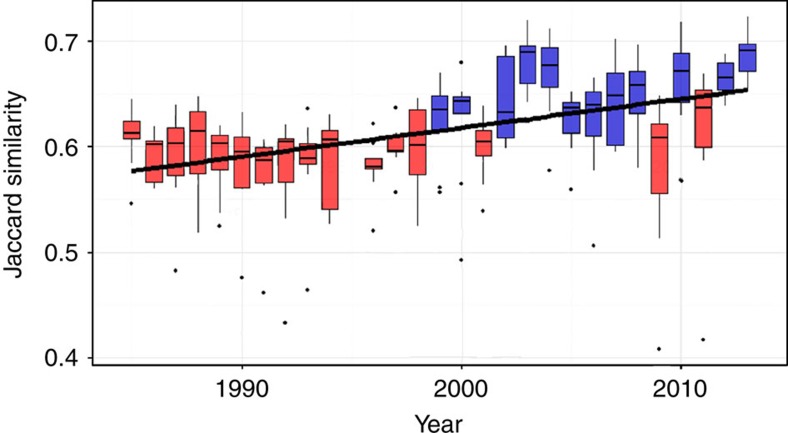
Box plots (median, quartiles, range and outliers) of pairwise similarities (Jaccard) between latitudinal bands in each year of the study. The trend line (OLS regression) is shown (*r*^2^=0.42, *P*<0.001). Years in which the mean pairwise similarity is below the overall mean are shown in red, and those above the mean in blue. (A similar pattern emerges if Bray–Curtis similarity is used. See Supplementary Figure 3).

**Figure 3:**
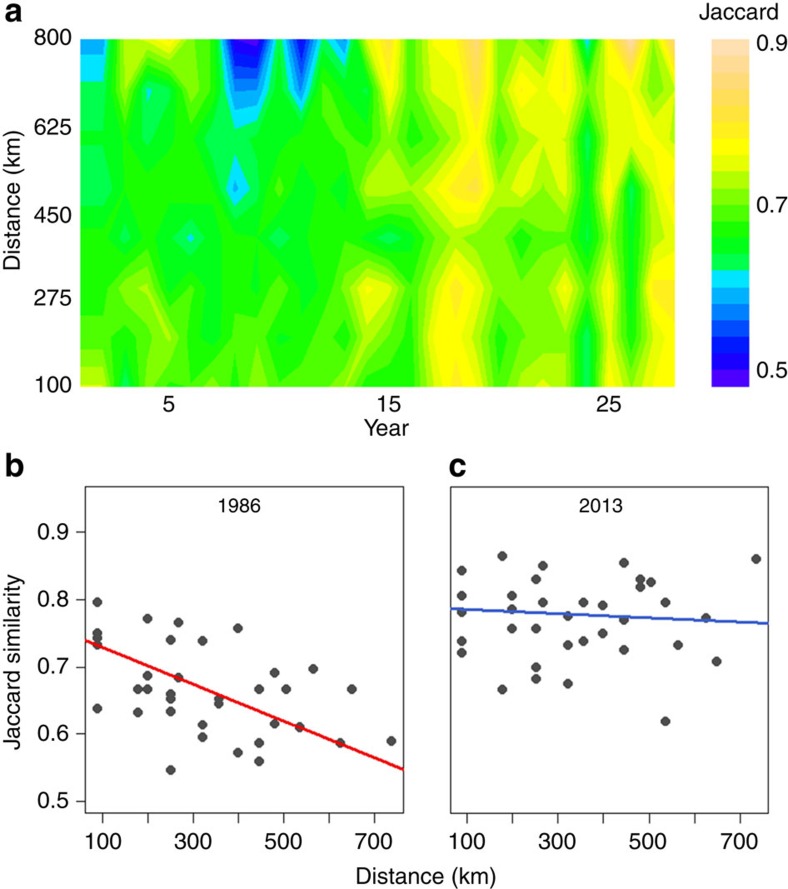
Similarity over space and time. (**a**) Contour plot illustrating the relationship between Jaccard similarity and geographical distance (between latitudinal bands) over the duration of the study. The scale bar is to the right of the plot. The plot highlights increasing similarity through time, and greater homogenization across distant localities. (bottom) Distance-decay plots for an early ((**b**) 1986) and late ((**c**) 2013) year in the study. Median slopes shown. Distances (km) are between latitudinal bands. Compositional similarity of more distant localities increases through time (See Supplementary Figure 4 for further analysis of distance decay).

It is possible for temporal turnover to be independent at each site, with no spatial signature. Instead, we found the temporal turnover depicted in Fig. 1 is at least partly explained by the species composition in the different latitudinal bands becoming progressively more similar over time (Fig. 2). Moreover, the distance-decay relationship in composition is less steep in later years, indicating that the northern and southern assemblages are now less differentiated from one another than they were three decades ago (Fig. 3 and Supplementary Fig. 4). The result of these progressive changes is spatial biotic homogenization. These analyses confirm the hypothesis of Dornelas *et al.*^6^ that biotic homogenization can lead to a pattern of constant local species richness but increasing change in species composition. As further support, an analysis of spatial β diversity partitioning^18^,^19^ reveals that turnover in species identity is a consistently stronger driver of community reorganization than change in species richness (Supplementary Fig. 5 and Supplementary Fig. 6). This result is in contrast to a recent meta-analysis^20^ which argued that changes in community similarity are driven by changes in species richness rather than changes in species composition.

### Water temperature

**Figure 4:**
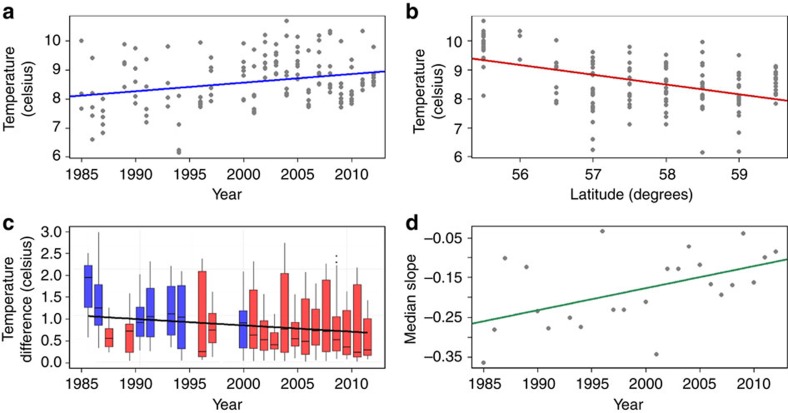
Similarity and temperature. (**a**) Mean oceanographic surface temperature (depth<10 m) data in the first quarter of the year for the study area, in relation to year. The plot shows an increase in mean temperature through time (OLS regression, *r*^2^=0.22, *n*=162). (**b**) Relationship between temperature and latitude ignoring year, indicating that, on average, water temperature is cooler in more northern localities. (**c**) Box plot (median, quartiles, range and outliers) of pairwise temperature differences between latitudinal bands (analogous to the plot in Figure 2). Years in which the mean pairwise difference is below the overall mean are shown in red, and those above the mean in blue. There is a significant negative correlation (Spearman *r*_s_=−0.46, *P*=0.026, *n*=23) between mean annual temperature difference (this plot) and mean annual Jaccard dissimilarity (1-Jaccard). This indicates that the reduction in temperature difference parallels the reduction in differences in community composition. (**d**) Median slope of the relationship between temperature and latitude in each year of the study showing the steepness of the temperature gradient is declining over time. This trend is significant (Spearman *r*_s_=0.6, *P*=0.001, *n*=27).

What forces are responsible for this widespread reorganization of marine fish assemblages? In this region of the North Atlantic, average ocean temperature has increased by c. 1 °C over the time frame of this study (Fig. 4a). Moreover, the south–north temperature gradient has decreased, so that the latitudinal bands in Fig. 1 are now thermally less differentiated than they used to be (Fig. 4d), meaning the northerly waters have increased in temperature more rapidly than the southerly waters. The mean annual pairwise difference in temperature between latitudinal bands (Fig. 4c) also declines through time. This decline mirrors the shift in community similarity indicating that, as water temperatures within the latitudinal bands converge over time, so too does the composition of the assemblages found there.

## Discussion

This investigation has documented rapid biotic homogenization in marine fish assemblages and argued that these community shifts are linked to climate. Other recent studies also highlight the potential of anthropogenic climate change^21^,^22^ to restructure marine communities. Climate velocity has been identified as a predictor of biotic change^14^. Our data show that these transformations are manifested over relatively small geographic areas and short time periods. It is likely that climate change acts in synergy with other anthropogenic stressors, such as pollution and over-exploitation^23^. However, although fishing has heavily impacted the North Atlantic for over a century, fishing effort (as reported landings and trawling hours) has not increased appreciably over the time period of this study^24^,^25^. Moreover, coastal human population densities in this region have remained low over the last three decades (for example, in 2011 mean human population density was 5.1 km^−2^).

Our perception of the natural state of marine communities is shaped by shifting baselines^26^. Contemporary accounts of Scottish waters in the eighteenth century attest to greater abundances of fish and sea mammals present at that time while drawing attention to the impacts that commercial fishing were having over 200 years ago^27^. The homogenization we document here is a continuation of the ongoing change in fish communities exposed to long-term anthropogenic pressures.

As we have shown, apparent constancy in widely used measures of α diversity (that is, lack of trends in local species richness) can mask enormous changes in an ecosystem. Local richness has, on average, been maintained over the duration of this study, with the same common species (including the Norway pout *Trisopterus esmarkii*, and Atlantic herring *Clupea harengus*) continuing to be overall dominants. However, our analyses reveal that groundfish communities off western Scotland are undergoing rapid reorganization. This change is driven by spatial homogenization, that is increasing similarity between locations 100 s of kms apart–possibly linked to climate change. Our data suggest that predicted changes in marine fish communities are already underway^6^,^17^. Biogeographical shifts in zooplankton species in the North East (NE) Atlantic^28^ provide further evidence that the pace of change is marine communities exceeds that in the terrestrial realm. This collapse of spatial β diversity (that is, increasing homogenization) is arguably a far greater and more pressing crisis than the loss of local species richness. As long as species are not globally extinct this homogenization is potentially reversible. However, this crisis is largely unrecognized, and adds to the challenges already facing marine biodiversity^23^.

## Methods

### Data

The Scottish groundfish survey takes place annually, in the seas to the north and west of Scotland (Supplementary Fig. 1). Sampling began in 1985. Trawling occurs in the first quarter of the year (January, February and March), and the same methodology has been used throughout. Each sample is a discrete trawl occurring at a precise geographical point within an International Council for the Exploration of the Seas (ICES) statistical rectangle (rectangles represent a 30′ latitude by 1° longitude grid cell). Sample number varies between rectangles in a given year. Species abundances are reported as catch per unit effort (that is the number of individuals per species caught during a 1 h trawl). Body size (standard length) is also recorded. We focus on the 35 rectangles (Supplementary Fig. 1) for which there are good community time series. A total of 131 taxa have been sampled in this study area over the 29 years of the survey. 126 of these taxa are finfish species, the remaining 5 macroinvertebrates (squid and macrocrustaceans). As 1992 and 1995 have substantially fewer samples than other years, we exclude them from our analyses. Species and temperature data were downloaded from the ICES portal (DATRAS Fish Survey Data ‘Scottish West Coast Survey For Commercial Fish Species 1985–2013’ (Available at https://datras.ices.dk, accessed on 2014)). Information on coastal human population density was obtained from National Records of Scotland, 2011 Census: Aggregate data (Scotland). UK Data Service Census Support. Downloaded from: http://infuse.mimas.ac.uk. This information is licensed under the terms of the Open Government Licence (http://www.nationalarchives.gov.uk/doc/open-government-licence/version/2) accessed on 2015.

### Analysis

To understand how the composition of these groundfish communities is changing through time, relative to latitude, we first assign the rectangles to nine 30′ latitudinal bands. We then compile a community time series for each latitudinal band. Sample rarefaction (as in ref. 6) ensures equal sampling effort across bands and is used in the calculation of temporal α diversity (species richness) and temporal β diversity (Jaccard similarity (presence/absence) and Bray–Curtis similarity (quantitative)). We first calculate similarity in relation to the start of the survey. Next, for each year, we compute the pairwise compositional similarity of these latitudinal bands. We also construct distance-decay plots (similarity plotted against geographic distance) for each year. Quantile regression is used to fit the median slope of these relationships as it is robust against outliers. β diversity partitioning^18^,^19^ enables us to compute the relative contributions of turnover and nestedness to community change. To do this we recalculate Jaccard similarity as Jaccard dissimilarity because β diversity partitioning conventionally focuses on dissimilarity.

Geographical distances (in km) are calculated using the point distance tool in ArcGIS. Analyses use R statistical software^29^. The vegdist function in the R package vegan^30^ is used to compute similarities. The quantreg package^31^ is used to fit median slopes, and the betapart package^19^ to partition β diversity into components of turnover and nestedness.

## Additional information

**How to cite this article:** Magurran, A. E. *et al.* Rapid biotic homogenization of marine fish assemblages. *Nat. Commun.* 6:8405 doi: 10.1038/ncomms9405 (2015).

## Supplementary Material

Supplementary Information Supplementary Figures 1-6 and Supplementary References
