# Supplementary material for: Rapid biotic homogenization of marine fish assemblages
Source: Nat Commun. 2015 Sep 24;6:8405. doi: 10.1038/ncomms9405 (PMC4598618; doi:10.1038/ncomms9405)
Supplement: Supplementary Information — Supplementary Figures 1-6 and Supplementary References [file ncomms9405-s1.pdf]

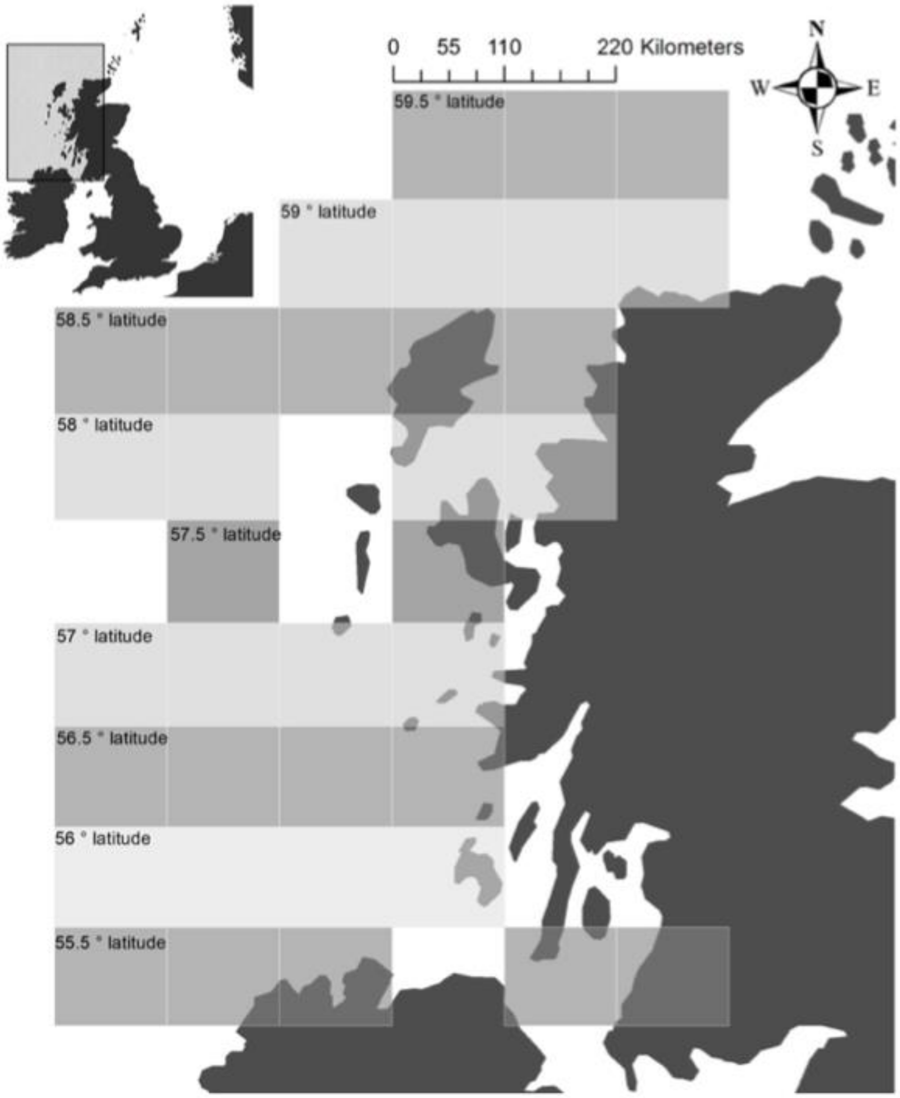

Supplementary Figure 1: Location of study area showing the 9 latitudinal bands and 35 ICES statistical rectangles

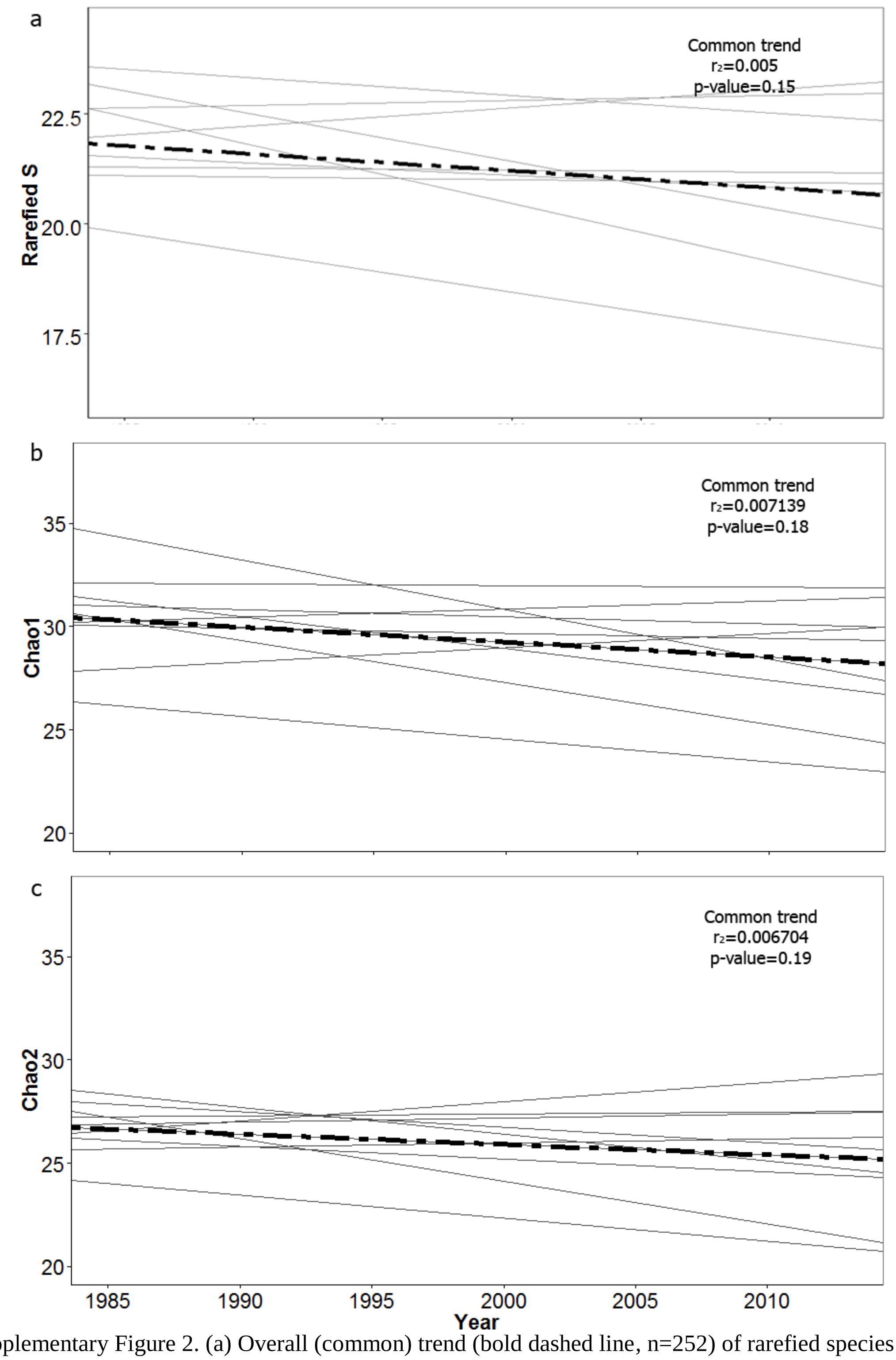

Supplementary Figure 2. (a) Overall (common) trend (bold dashed line, n=252) of rarefied species richness in the nine latitudinal bands (OLS regression). Grey lines show trends for each latitudinal band, as in Figure 1. (b) Overall (common) trend (n=252) of extrapolated species richness (Chao 1) in the nine latitudinal bands. (b) Overall (common) trend (n=252) of extrapolated species richness (Chao 2) in the nine latitudinal bands.

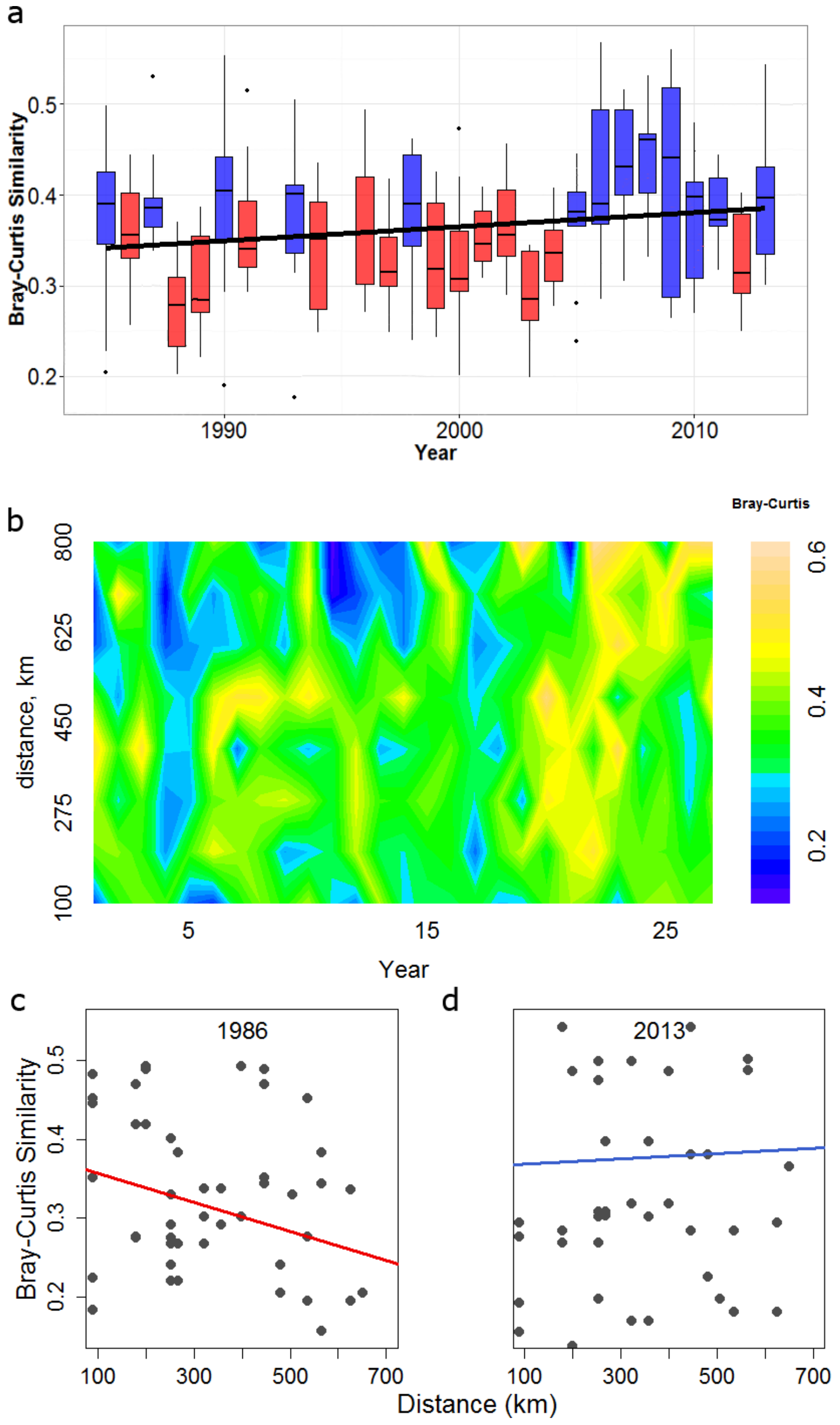

Supplementary Figure 3 (a) Box plots (median, quartiles, range and outliers) of pairwise similarities (Bray-Curtis) between latitudinal bands in each year of the study. The trend line (OLS regression) is shown (r^2=0.12, n=252). Years in which the mean pairwise similarity is *below* the overall mean are coloured red, and those *above* the mean are coloured blue. (b) Contour plot illustrating the relationship between Bray-Curtis similarity and geographical distance (between latitudinal bands) over the duration of the study. The scale bar is the right of the plot. Plot highlights the increasing similarity through time, and the increasingly greater homogenization across distant localities. (c&d) Distance decay plots for an early (c: 1986) and late (d: 2013) year in the study. Median slopes shown. Distances (km) are between latitudinal bands. Compositional similarity of more distant localities increases through time.

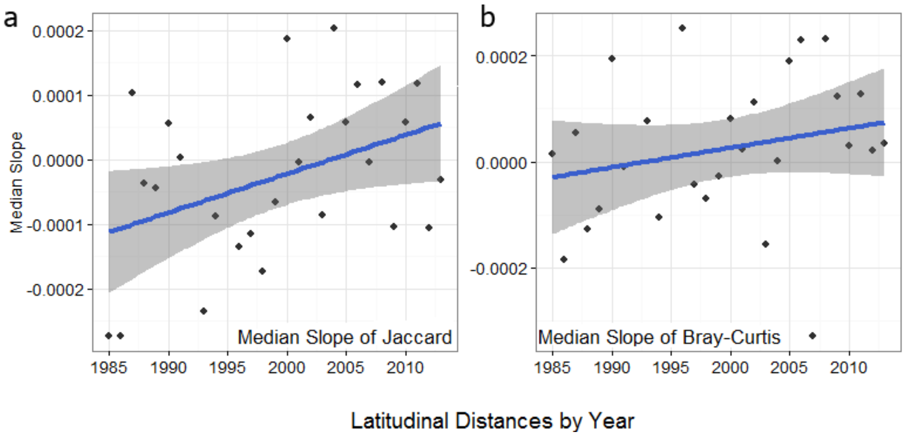

Supplementary Figure 4 (a): Median slopes of distance decay plots of the relationship between Jaccard similarity and latitudinal distance (latitudinal bands). (b) Median slopes of distance decay plots using Bray-Curtis similarity. In both cases, as shown by the OLS regression line (blue) and 95% confidence band (grey), distance decay slopes become shallower with time.

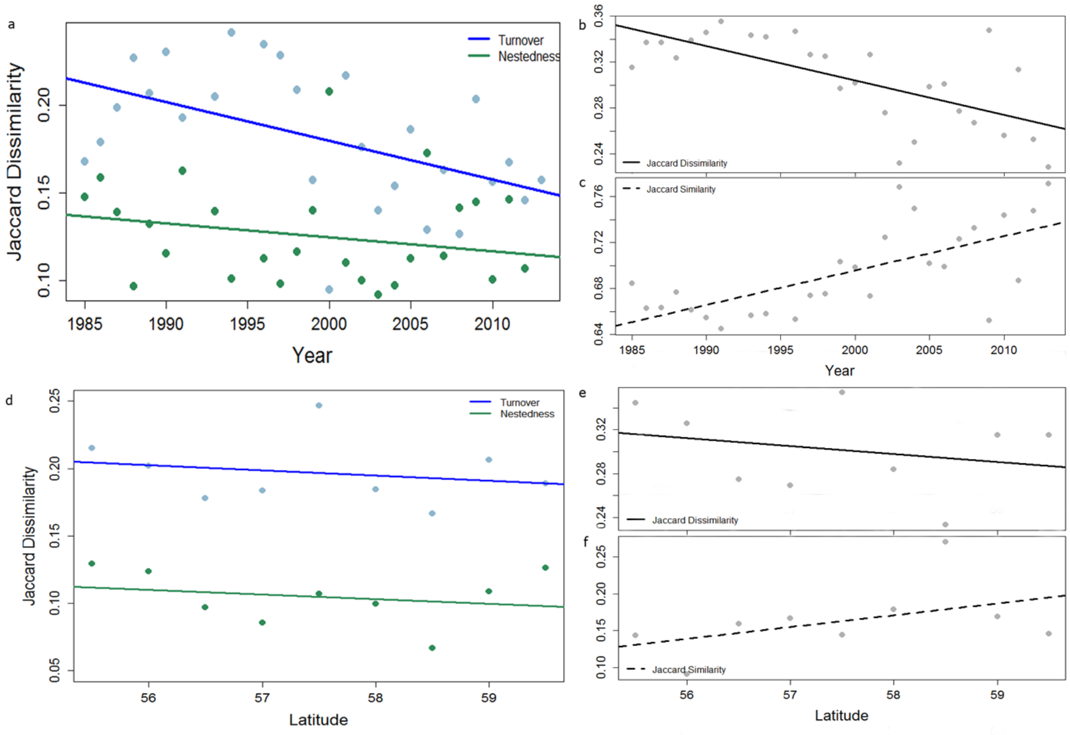

Supplementary Figure 5: (a): Mean spatial Jaccard dissimilarity (pairwise comparison: latitudinal bands), partitioned into turnover and nestedness components, in each year of the study (OLS regression lines shown) [1,2]. Nestedness captures the change due to shifts in species richness while turnover is the component of the measure linked to change in species identity. Turnover consistently accounts for a higher fraction of the observed Jaccard dissimilarity. (right) β diversity partitioning conventionally uses Jaccard dissimilarity (b) which is the obverse of similarity (c, and as in Figure 2) . (d): Mean temporal Jaccard dissimilarity (pairwise comparison: latitudinal bands), partitioned into turnover and nestedness components, in each latitudinal band. Turnover is, again, most important. (d) Jaccard dissimilarity and (e) similarity plots for temporal turnover. Further support for the conclusion that β diversity, rather than α diversity, is driving community change is provided by Supplementary Figure 6.

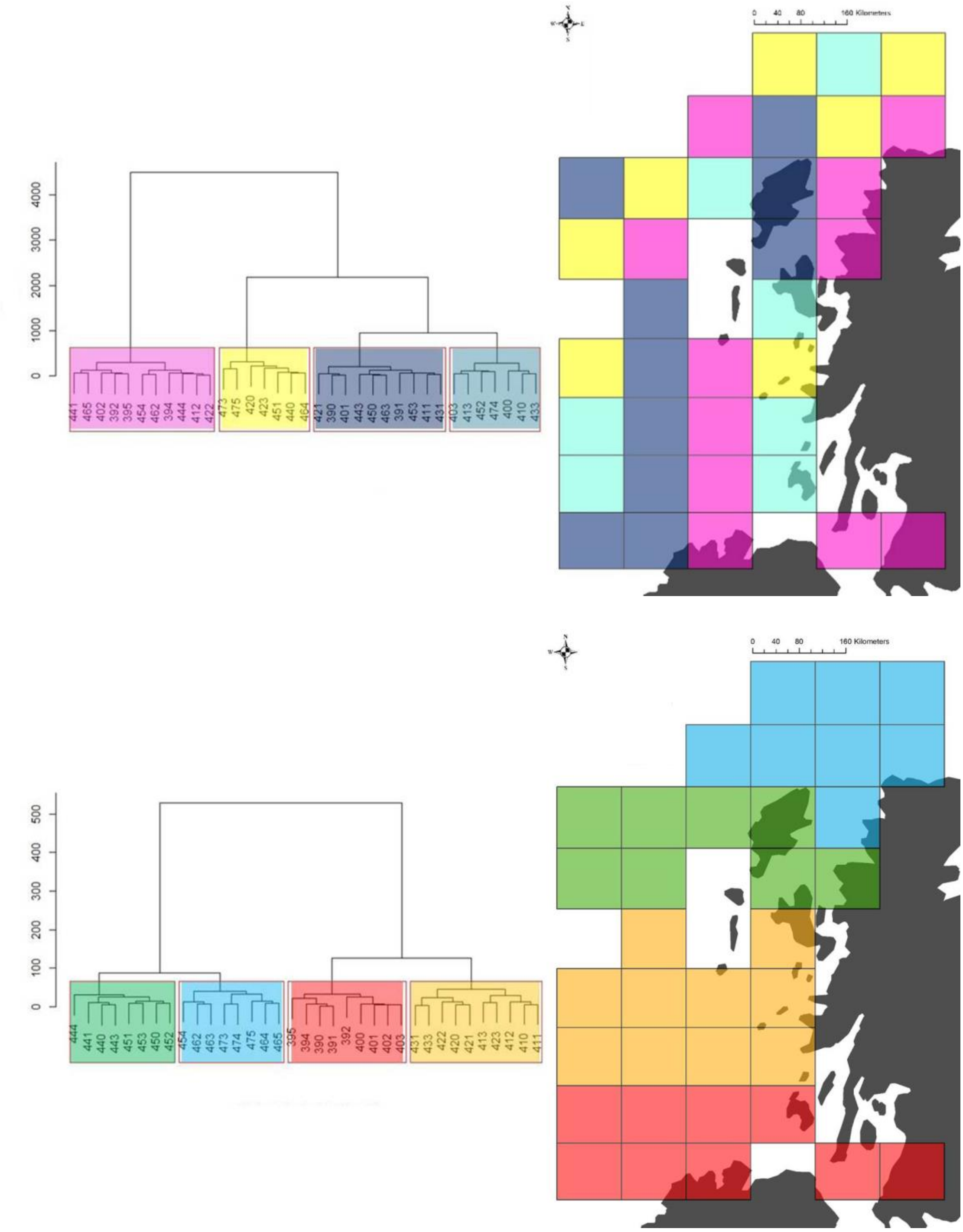

Supplementary Figure 6

Cluster analysis can be used to examine the relationship between temporal diversity and latitude. This investigation, using ICES rectangles, provides further support for a latitudinal signal in β diversity, but not in α diversity. We first plot the relationship between α diversity and time, for each rectangle, and then fit the median slope to this plot. Three temporal α diversity metrics are used: rarefied S, numerical abundance (as CPUE) and median body size. The analysis is repeated using 4 temporal β diversity measures (in all cases calculated relative to the baseline (start date) within each rectangle) are used: Jaccard, Morisita-Horn, Bray-Curtis, and Chao (see [3]). Cluster analysis is based on Euclidian distance and uses Ward’s method. In each case the analysis suggests 4 clusters. The geographic locations of the four clusters are indicated on the map by colour coding.

Supplementary References

1 Baselga, A. Partitioning the turnover and nestedness components of beta diversity. *Global Ecology and Biogeography* **19**, 134-143, doi:10.1111/j.1466-8238.2009.00490.x (2010).

2 Baselga, A. *et al.* betapart: Partitioning beta diversity into turnover and nestedness components. R package version 1.3. http://CRAN.R-project.org/package=betapart. (2013).

3 Dornelas, M. *et al.* Assemblage time series reveal biodiversity change but not systematic loss. *Science* **344**, 296-299 (2014).
